# The facile synthesis and bioactivity of a 3D nanofibrous bioglass scaffold using an amino-modified bacterial cellulose template

**DOI:** 10.1039/c8ra00352a

**Published:** 2018-04-18

**Authors:** Cuilian Wen, Yun Hong, Junru Wu, Lijin Luo, Yimei Qiu, Jianxia Ye

**Affiliations:** College of Materials Science and Engineering, Fuzhou University, Key Laboratory of Eco-materials Advanced Technology (Fuzhou University), Fujian Province University Fuzhou 350116 China clwen@fzu.edu.cn; Fujian Provincial Key Laboratory of Screening for Novel Microbial Products, Fujian Institute of Microbiology Fuzhou 350007 China luolijin@sina.com

## Abstract

Porous bioglass (BG) scaffolds are of great importance in tissue engineering because of their excellent osteogenic properties for bone regeneration. Herein, we reported for the first time the use of amino-modified bacterial cellulose (NBC) as a template to prepare a three-dimensional (3D) nanofibrous BG scaffold by a facile modified sol–gel approach under ultrasonic treatment. The results suggested that the amino groups on the BC template could effectively promote the absorption of the deposited CaO and SiO_2_ precursors, and the as-obtained BG scaffold showed a 3D interconnected porous network structure consisting of nanofibers with a diameter of about 20 nm. Furthermore, the as-obtained BG scaffold showed very good bioactivity after being immersed in SBF for 7 days. This research provides a facile and efficient way to prepare a nanofibrous BG scaffold with 3D porous structure, which can be used as a promising candidate for biomedical applications.

## Abstract 2

A nanofibrous BG scaffold with a high quality 3D porous interconnected structure has been prepared *via* a facile modified sol–gel approach using amino-modified bacterial cellulose as the template.

## Graphical abstract

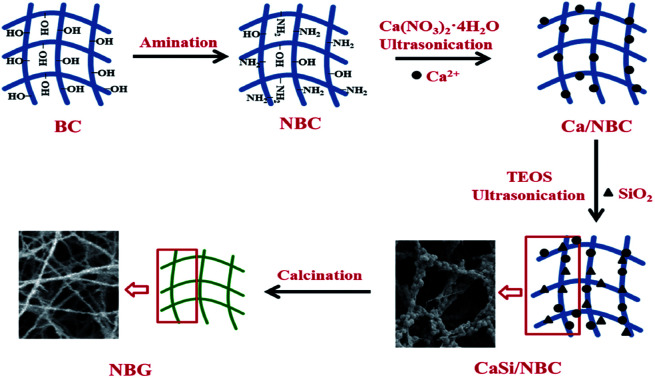

## 1. Introduction

Bioactive glasses have attracted an increasing amount of interest in orthopedic and dental fields as well as bone tissue engineering due to their remarkable bioactivity, biocompatibility, and osteogenic and angiogenic effects.^1–4^ Moreover, it is generally agreed that highly porous microstructures with large surface areas can stimulate cell growth.^5,6^ Therefore, in most of the scaffold preparation methods used for tissue engineering, there is a main requirement to have an interconnected porous network to enable the transportation of oxygen, nutrients and exudates as well as scaffold degradation products.^7^ Considerable efforts have been focused on the improvement of BG scaffold preparation strategies for obtaining aptly designed structures and modified surface properties. For bone regeneration, BG scaffolds with interconnected 3D pore structures can provide an appropriate environment in which cells and tissues can dwell.^8,9^ In addition, researchers have found that in natural bone tissues, the extracellular matrix (ECM) consists of collagen nanofibers with a diameter of 50–500 nm, which can largely control the cell behavior and function.^9,10^ Therefore, it is very important to fabricate a scaffold with interconnected pores and a 3D architecture that can mimic the nanofibrous ECM structure and can possess sufficient pores to provide space for tissue ingrowths and nutrient transport to the center of the regenerated tissues.^11^

To date, such nanofibrous scaffolds were produced using a range of different techniques including through drawing,^12^ template synthesis,^13^ phase separation^14,15^ and self-assembly.^1,16–20^ These techniques can produce scaffolds with various morphologies and porosities. For instance, Liverani *et al.*^21^ creatively combined sponge replica, freeze-drying and electrospinning methods to prepare a multilayered stratified composite scaffold. Bretcanu *et al.*^5^ obtained biodegradable polymeric nanofibers with an average diameter of 200 nm by electrospinning different polymers onto sintered 45S5 Bioglass®-based glass-ceramic pellets. In addition, Echezarreta-López *et al.*^22^ used the laser-spinning technique to produce Zn–Sr-doped BG nanofibers with antibacterial properties. It is noteworthy that the electrospinning method has gained a lot of attention due to the simplicity of the nanofiber preparation process. However, electrospun nanofibrous scaffolds lack the ability to produce the required 3D macroporous geometry, and the fiber diameters are often in the scale of hundreds of nanometers, which cannot mimic the complexity of natural ECM.^23^

In recent years, bacterial cellulose (BC) has also been widely investigated for tissue engineering because it possesses a natural refined 3D nanofibrous network, which has a shape similar to that of the collagen nanofibers found in natural tissues.^10,24–27^ Researchers have developed artificial blood vessels^28^ and cartilages^29^ with the help of BC, which has a good fiber network, biocompatibility, high water retention and high tensile strength. Very recently, Luo *et al.*^30^ prepared 3D nanofibrous BG scaffolds composed of fibers of about 30 nm diameters using BC as a template *via* a sol–gel approach. However, it is worth pointing out that the fabrication cycle of the BG scaffold lasted for nearly a week, and the as-obtained BG scaffolds were flattened to a certain extent; thus, Luo *et al.* could not construct a good 3D interconnected porous structure. Therefore, improving the preparation efficiency and controlling the quality of the 3D porous structure of the BG scaffold is very important. However, in literatures, there are very few studies reporting the use of a BC template to prepare and control the quality of a 3D porous BG scaffold. This has prompted us to carry out an extensive and systematic investigation on this topic, which is of great interest and importance.

From our research, it is found that the adsorption of CaO and SiO_2_ precursors on a BC template is a very important step during the preparation of a BG scaffold. However, it is known that it is very difficult to take advantage of hydroxyl groups (–OH) to adsorb cations.^17^ Therefore, improving the absorption capacity of BC fibers towards CaO and SiO_2_ is an effective way to improve the preparation efficiency of BG nanofibers. According to our previous research, amino-modified BC fibers can enhance their adsorption abilities towards metal cations in wastewater.^31^

In this research, amino-modified BC has been used as the template, and a facile modified sol–gel approach under ultrasonic treatment has been used to prepare a nanofibrous BG scaffold with a high quality 3D porous structure, which can effectively solve the above-mentioned problems. The microstructures and phase constituents of the as-obtained BC templates and BG scaffolds are characterized by XRD, FT-IR, SEM, EDS and BET analyses. The effects of the amino-modified BC template on the formation of 3D nanofibrous BG scaffolds have been investigated. Moreover, the bioactivity has been studied using an immersion test in SBF solution.

## 2. Experimental

### 2.1 Materials

BC obtained from *Acetobacter xylinum* culture was provided by the Fujian Institute of Microbiology (Fujian, China). Ceric ammonium nitrate (CAN) was purchased from Shanghai Macklin Biochemical Co., Ltd. (Shanghai, China). All other reagents were purchased from Sinopharm Chemical Reagent Co., Ltd. (Beijing, China). All reagents were of analytical grade.

### 2.2 Preparation of BC and amino-modified BC scaffolds

The BC membranes were cut into pieces with dimensions of about 20 mm × 20 mm × 2 mm and purified in 0.5 M NaOH solution at 100 °C for 2 h; then, they were washed with a sufficient amount of deionized water to neutrality to remove the residual bacterial cells.^31^ Then, the BC membranes were placed in *tert*-butyl alcohol and freeze-dried to obtain the BC scaffolds, which were designated as BC. Amino-modified BC scaffolds were prepared by grafting glycidyl methacrylate (GMA) followed by its reaction with ethylenediamine (EDA).^32,33^ First, the BC membranes were immersed in 160 mL of deionized water. Then, the temperature was increased to 35 °C, and N_2_ was purged through the reaction mixture for 30 min to remove any dissolved O_2_. Twenty mL of 0.1 M CAN (dissolved in 1 M HNO_3_) solution was added, and the resulting mixture was continuously stirred for 15 min. Then, 4 mL of GMA was slowly added dropwise over 30 min, and the reaction was conducted for 2 h. The resulting GMA-grafted BC was washed several times with deionized water and ethanol and then freeze-dried. The freeze-dried BC was dispersed in a mixture composed of 75 mL of EDA and 50 mL of deionized water. This mixture was kept at 80 °C under stirring for 2 h and then washed several times with deionized water and ethanol until a neutral pH was attained. The as-obtained product was placed in *tert*-butyl alcohol and freeze-dried to obtain the amino-modified BC scaffold, which were designated as NBC.

### 2.3 Preparation of the BG scaffold

The BG precursors were deposited on the NBC scaffold after being immersed in an ethanol solution of Ca(NO_3_)_2_·4H_2_O and tetraethyl orthosilicate (TEOS) solution under ultrasonic treatment using an ultrasonic water bath operated at a flexed frequency of 40 kHz and 130 W average ultrasonic power (Transsonic Digitals-KQ2200DE Kunshan, China). The NBC template was immersed in 0.2 M Ca(NO_3_)_2_·4H_2_O solution for 90 min in a glass baker placed in the ultrasonic bath for pre-calcification; then, it was carefully rinsed with deionized water and immersed in a silica sol precursor solution for another 90 min of ultrasonication. The silica sol precursor was prepared using 1 mL of TEOS with 40 mL of ethanol, and aqueous ammonia was added to control the pH to 2. The product was then rinsed with deionized water and placed in *tert*-butyl alcohol to be freeze-dried, and the obtained product was denoted as CaSi/NBC. Finally, CaSi/NBC was calcined at 700 °C for 3 h to obtain the BG scaffold, which was denoted as NBG. For comparison, the product prepared using pure BC as a template was used as the control group, which were respectively denoted as CaSi/BC and BG.

**Fig. 1:**
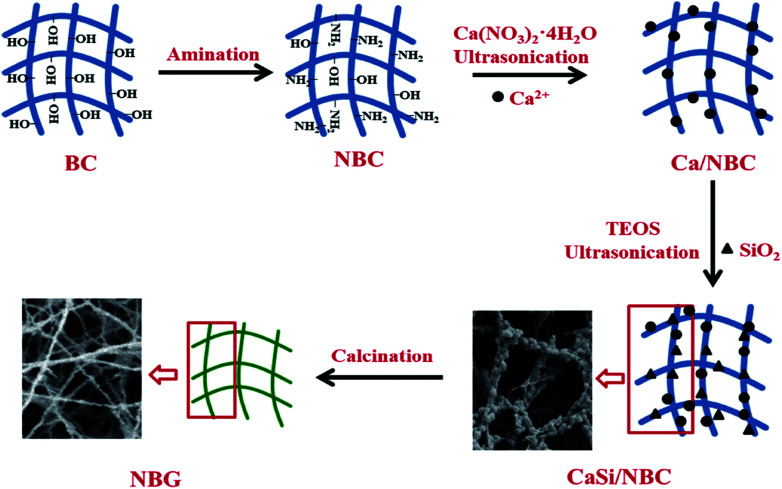
Schematic representation for the fabrication of nanofibrous NBG scaffold.

A schematic representation for the fabrication of nanofibrous NBG scaffold is illustrated in Fig. 1. The NBC scaffold was prepared through the grafting of GMA followed by a reaction with EDA, and this was followed by pre-calcification for absorbing Ca^2+^ under ultrasonication. Afterwards, the pre-calcified NBC was immersed in a silica sol precursor solution under another ultrasonic treatment to adsorb the silica precursors onto the surface of NBC nanofibers. The ionic interactions of the amino groups (–NH_2_) to replace the hydroxyl groups (–OH) of NBC could act as active sites,^31^ which caught more Ca^2+^ cations during the pre-calcification process. Thus, the uniform distribution of –NH_2_ groups on the NBC nanofibers and ultrasonic treatment during the sol–gel process could promote an even absorption and dispersion of the CaO and SiO_2_ precursors on the surface of the NBC nanofibers. Finally, the 3D nanofibrous NBG scaffold was obtained by calcinating CaSi/NBC when the BC template burned out during the calcination process. The morphologies of the as-obtained CaSi/NBC and NBG scaffolds are shown in Fig. 1.

### 2.4 Characterization

The phase composition of the as-obtained BC, NBC, CaSi/BC, CaSi/NBC, BG, NBG samples and the samples obtained after immersion in SBF were analyzed by XRD (Miniflex 600, Rigaku, Japan) using nickel-filtered Cu-Kα radiation (*λ* = 0.15418 nm) operated at 40 kV and 15 mA. Diffraction patterns were generated between the 2*θ* values of 10–80° at a step increment of 0.01° and scanning speed of 4° min^−1^. The morphology and composition of the as-prepared samples were investigated using SEM (SUPRA-55, Zeiss, Germany) with EDS analysis. The FT-IR spectra of the samples were recorded using a FTIR spectrophotometer (Bruker T27, Germany) between 4000 and 400 cm^−1^ using the KBr pellet technique at room temperature. The TGA analysis (Q600, Switzerland) was performed from room temperature to 700 °C at a heating rate of 10 °C min^−1^ under a steady flow of nitrogen at 20 mL min^−1^. The specific surface areas and pore size distributions of the NBC and NBG samples were determined using the Brunauer–Emmett–Teller (BET) nitrogen adsorption–desorption isotherms and Barrett–Joyner–Halenda (BJH) analysis (3flex, Micromeritics, USA).

### 2.5 Immersion test

An immersion test in SBF was used to study the bioactivity of the nanofibrous NBG scaffolds. As reported previously,^34^ the SBF solution was prepared by dissolving NaCl, NaHCO_3_, KCl, K_2_HPO_4_·3H_2_O, MgCl_2_·6H_2_O, CaCl_2_·H_2_O, and Na_2_SO_4_ in deionized water with ion concentrations of Na^+^ (142.0 mM), K^+^ (5.0 mM), Ca^2+^ (2.5 mM), Mg^2+^ (1.5 mM), HCO_3_^−^ (4.2 mM), HPO_4_^2−^ (1.0 mM), SO_4_^2−^ (0.5 mM), and Cl^−^ (147.8 mM). The solution was buffered at pH 7.4 using tris(hydroxymethyl) aminomethane ((CH_2_OH)_3_CNH_2_) and 1 M HCl at 37 °C. The NBG samples were soaked in the SBF solution at 37 °C for 1, 3, and 7 days. The immersed solution was replaced every 2 days to maintain the concentration of the ions. The morphology and phase constituents were investigated using XRD, SEM and EDS analyses.

## 3. Results and discussion

### 3.1 Morphological evaluation

**Fig. 2:**
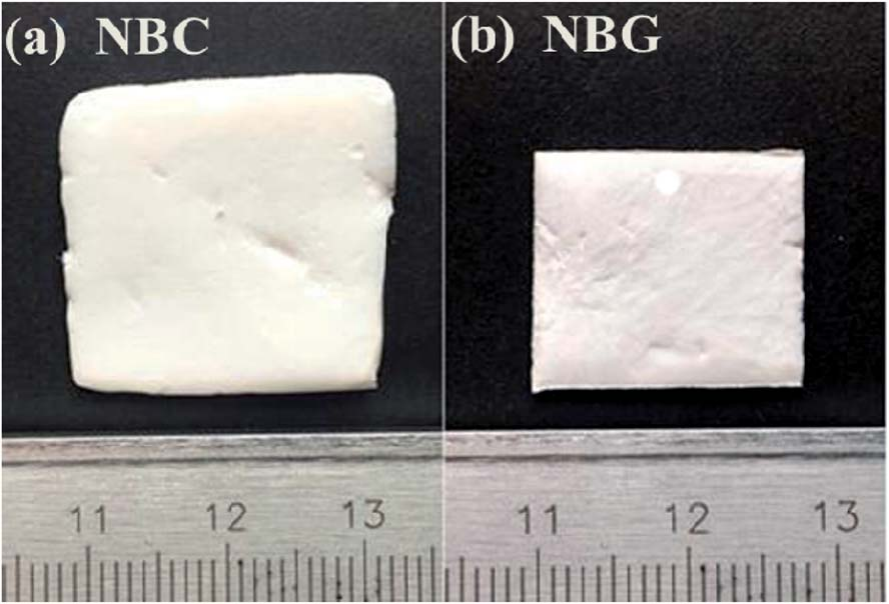
Macro-photographs of NBC and NBG scaffolds.

**Fig. 3:**
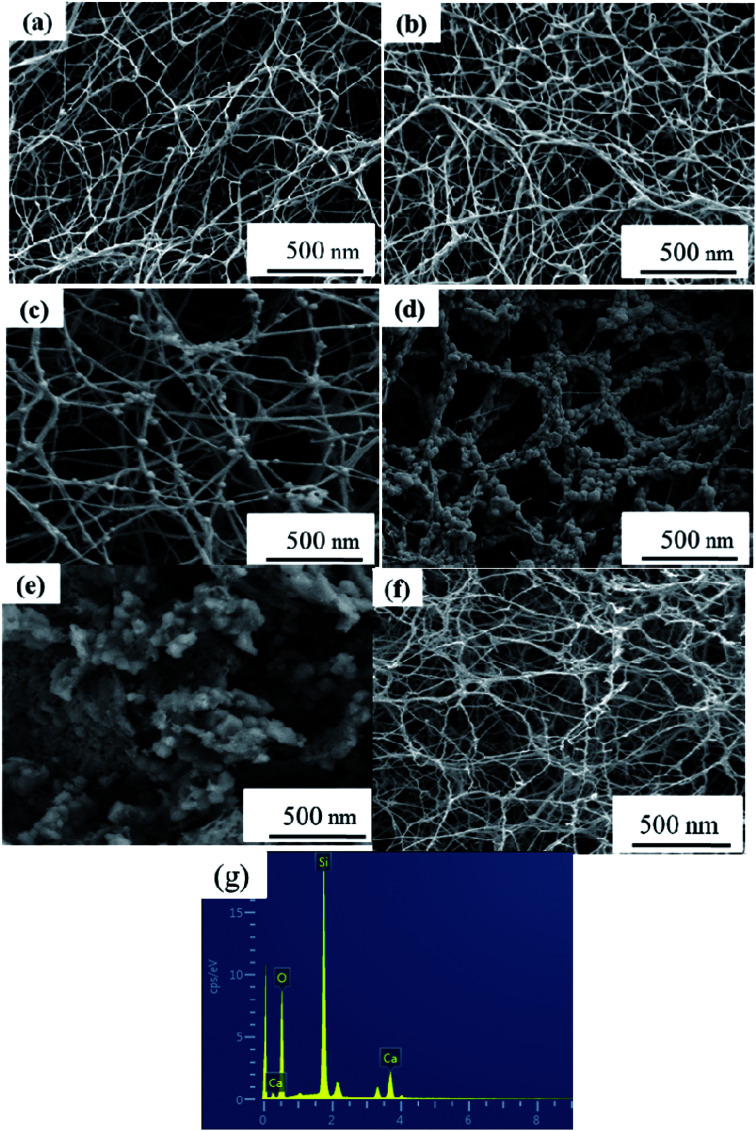
SEM images of (a) BC, (b) NBC, (c) CaSi/BC, (d) CaSi/NBC, (e) BG and (f) NBG scaffolds, and (g) EDS profile of NBG scaffold as shown in figure (f).

Macro-photographs of the NBC and NBG scaffolds are shown in Fig. 2. The photographs show that a complete NBG scaffold can be successfully prepared using this modified sol–gel method under ultrasonic treatment. When compared with the size of NBC scaffold, the size of the NBG scaffold is reduced by 1/5, indicating that due to the combustion of BC during the calcination process, the volume of the as-formed NBG scaffold contracts to a certain extent. The SEM images of the morphologies of the BC, NBC, CaSi/BC, CaSi/NBC, BG and NBG scaffolds are shown in Fig. 3a–f. The SEM images show that BC presents a nanofiber network with an average diameter of 30 nm (Fig. 3a). After grafting the –NH_2_ groups onto BC, the NBC scaffold is composed of uniform nanofibers, and the diameter of the fibers is not significantly larger than that of BC fibers as shown in Fig. 3b. Both BC and NBC show a similar 3D porous interconnected network structure, and the average pore size of the NBC scaffold is slightly smaller than that of the untreated BC sample, which may be related to the amination process of BC. As for the CaSi/NBC sample, there are a substantial number of particles homogeneously deposited on the surface of the NBC nanofibers after being immersed in the BG precursor solution under ultrasonic treatment. The average diameter of CaSi/NBC sample nearly reaches 68 nm, and the average pore size increases when compared with that of NBC as shown in Fig. 3b and d. In contrast, there are only few particles deposited on the surface of the pure BC fibers in the CaSi/BC sample, and the diameters of some CaSi/BC fibers slightly increase compared with those of the untreated BC fibers, which can be caused by the CaO and SiO_2_ precursors covered on the surface of BC fibers as shown in Fig. 3a and c. After calcination at 700 °C, it is found that the NBG scaffold shows a relatively good 3D interconnected porous network structure consisting of uniform and continuous fibers with an average diameter of about 20 nm as shown in Fig. 3f. When compared with the average pore size of CaSi/NBC, the average pore size of the NBG scaffold slightly contracts due to the combustion of BC during the calcination process. The as-obtained NBG scaffold shows relatively large surface area, and its structure is similar to that of ECM *in vivo*, which can provide an appropriate pore space for tissue ingrowth and nutrient transport to the regenerated tissues.^35^ In contrast, the BG sample without amination shows particle clusters and no fiber morphology, which can be due to the low activity of –OH groups on the surface of the BC fibers to adsorb enough Ca cations.^19^ Although the pre-calcification process is carried out under ultrasonic treatment for the pure BC sample, the enhancement in the chemical activity of the –OH groups is not very significant, and the CaO/SiO_2_ precursors deposit in lower amounts and in an extremely inhomogeneous manner. Therefore, an intense diffusion probably occurs when the CaSi/BC sample is calcined, which contributes to the breaking of fibers and partial agglomeration. These results suggest that the –NH_2_ groups can replace the –OH groups on the surfaces of the amino-modified BC fibers and act as active sites to catch more Ca^2+^ cations from the Ca(NO_3_)_2_ solution. Then, the incorporated Ca^2+^ can bond with a large quantity of SiO_2_ colloid particles present on the NBC nanofibers to form CaSi/NBC scaffolds as shown Fig. 3d.^36^ Meanwhile, the ultrasonic treatment during the sol–gel process can promote an even absorption and dispersion of the CaO and SiO_2_ precursors onto the surface of the NBC nanofibers, which is quite beneficial for the 3D structure formed when the BC template is burned out during the calcination process. The EDS profile of the NBG scaffold is shown in Fig. 3g and confirms the distribution of the Ca, Si and O elements in the NBG scaffold.

**Fig. 4:**
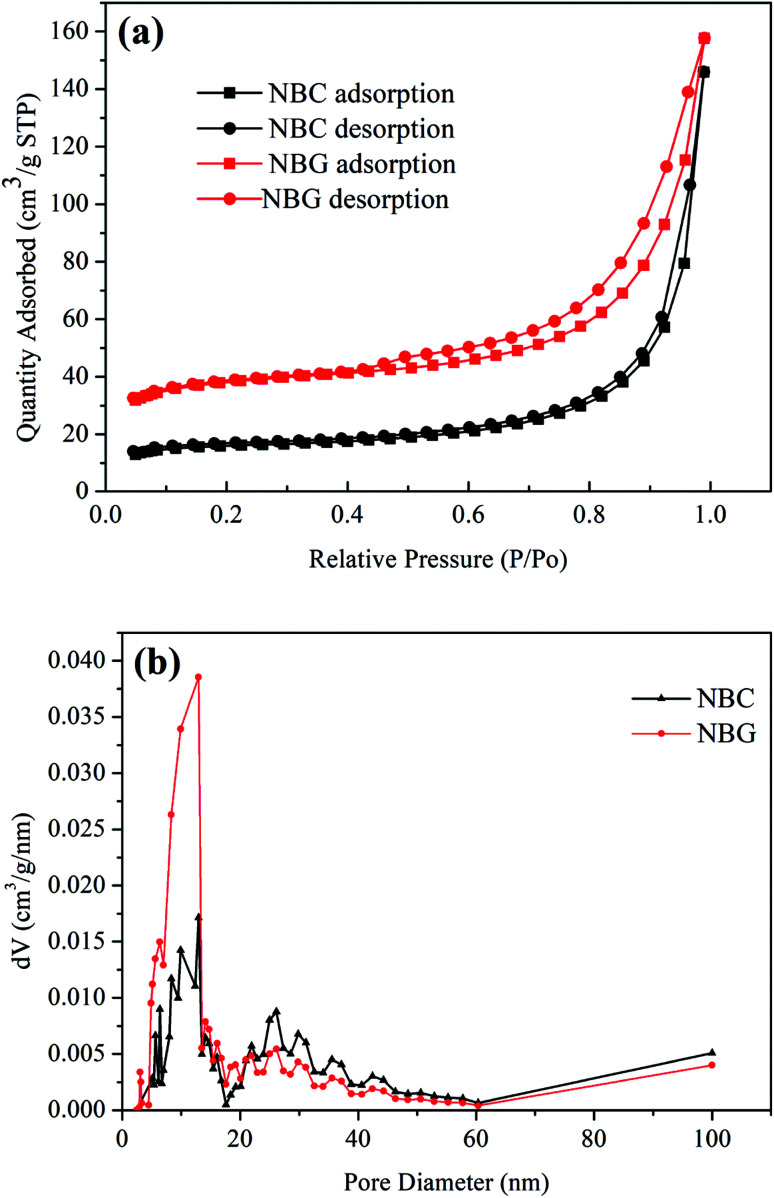
(a) Nitrogen adsorption/desorption isotherms and (b) pore size distributions of NBC and NBG scaffolds.

It is well accepted that the pore size of a scaffold can affect cell adhesion, cell proliferation and directional growth.^37,38^ The nitrogen adsorption/desorption isotherms and pore size distribution of the NBC and NBG scaffolds are shown in Fig. 4. According to the BDDT classification, all the isotherms shown in Fig. 4a are type IV hysteresis loops, which is typical for mesoporous materials. Quantitatively, the BET surface area, pore size and pore volume observed for the NBC and NBG scaffolds are summarized in Table 1. It is clearly seen that compared with the NBC scaffold, the NBG scaffold has a higher BET specific surface area, lower mesopore size and pore volume with values of 144.60 m^2^ g^−1^, 16.58 nm and 0.21 cm^3^ g^−1^, respectively. Fig. 4b shows the pore size distributions of the corresponding NBC and NBG scaffolds. The data are derived from their desorption curves using the BJH analysis. The curve for NBC shows a flat pore size distribution ranging from 5 to 45 nm. In contrast, the curve for NBG shows a pore size distribution ranging from 3 to 30 nm along with a very sharp volume peak at 13 nm. These results could be due to the BC combustion during the calcination process, contracted volume of NBG scaffold and decreased pore size, resulting in an increase in the specific surface area.

**Table 1:** The BET specific surface area, pore size and pore volume for NBC and NBG scaffolds

Sample	Specific surface area (m^2^ g^−1^)	Pore size (nm)	Pore volume (cm^3^ g^−1^)
NBC	60.33	24.51	0.22
NBG	144.60	16.58	0.21

### 3.2 FTIR spectroscopy analysis

**Fig. 5:**
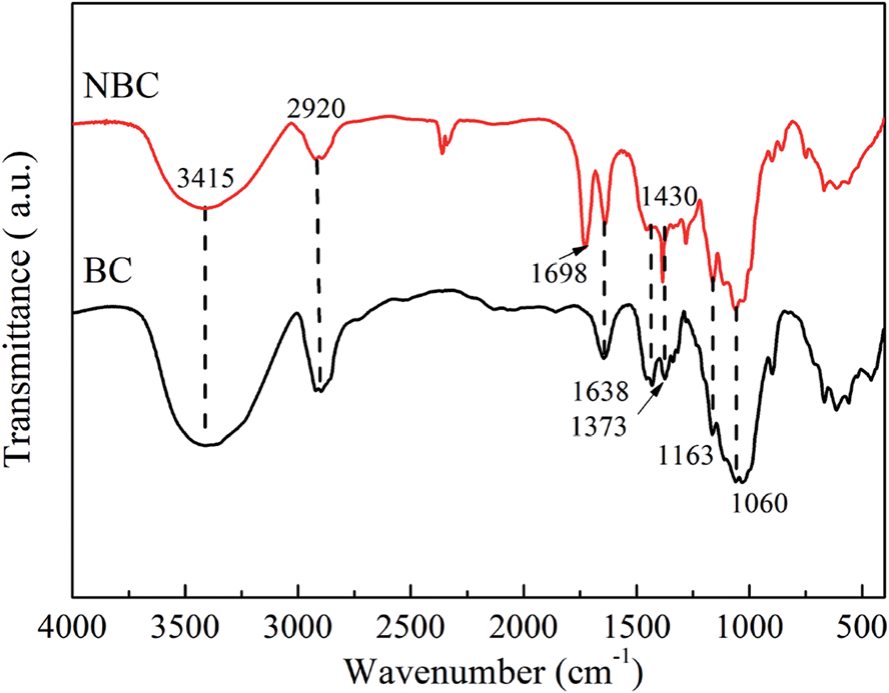
FT-IR spectra of BC and NBC samples.

FTIR analysis was performed to characterize the molecular composition and structures of the BC and NBC samples. Fig. 5 presents the FTIR spectra for BC and NBC samples. For the FTIR spectra of the BC sample, the characteristic peaks were respectively observed at 1638 cm^−1^ (carbonyl amide group), 1373 cm^−1^ (C–H bending), 1163 cm^−1^ (C–O–C stretching) and 1060 cm^−1^ (C–O stretching).^39,40^ The bands between 3200–3600 cm^−1^ were attributed to the vibrations of intermolecular and intramolecular hydrogen bonding, representing the –OH groups of cellulose.^41^ The presence of –NH_2_ groups in the NBC sample was confirmed by the characteristic peak at 1698 cm^−1^, which was ascribed to the –NH_2_ bending vibrations and indicated that the –NH_2_ groups had been successfully introduced onto the NBC structure. In fact, the –NH_2_ groups also showed stretching bands between 3200–3600 cm^−1^, which were overlapped by the stretching and bending vibrations of –OH groups on the surface of NBC.^42^

### 3.3 XRD analysis

**Fig. 6:**
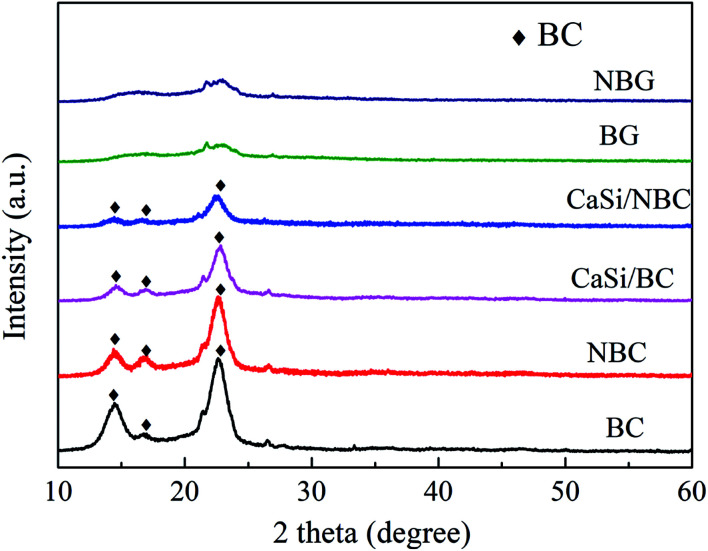
XRD patterns of BC, NBC, CaSi/BC, CaSi/NBC, BG and NBG samples.

The XRD patterns of the BC, NBC, CaSi/BC, CaSi/NBC, BG and NBG samples are shown in Fig. 6. It is clearly seen that the BC, NBC, CaSi/BC and CaSi/NBC samples have similar diffraction peaks, and the characteristic peaks at around 14.5°, 16.7°, and 22.7° can be assigned to the (1 −1 0), (1 1 0), and (2 0 0) crystal planes of the typical cellulose I pattern (PDF#03-0289); these results are in accordance with the results of previous studies.^43,44^ The broad diffraction peaks observed are due to the BC being a less crystalline material. The grafting of –NH_2_ groups does not show any influence on the crystallinity of NBC, which can be due to the small amount and the well-dispersed –NH_2_ groups on the BC surface.^45^ In addition, with the introduction of the CaO and SiO_2_ precursors under ultrasonic treatment, the crystallinity of BC is reduced in the CaSi/BC and CaSi/NBC samples. Moreover, BG is confirmed to be amorphous because no obvious diffraction peaks appear in the XRD spectra recorded for the BG and NBG samples, illustrating that the CaSi/BC and CaSi/NBC nanofibers have undergone a glass transition and formed an amorphous phase after being calcined at 700 °C.^46,47^

### 3.4 TGA analyses

**Fig. 7:**
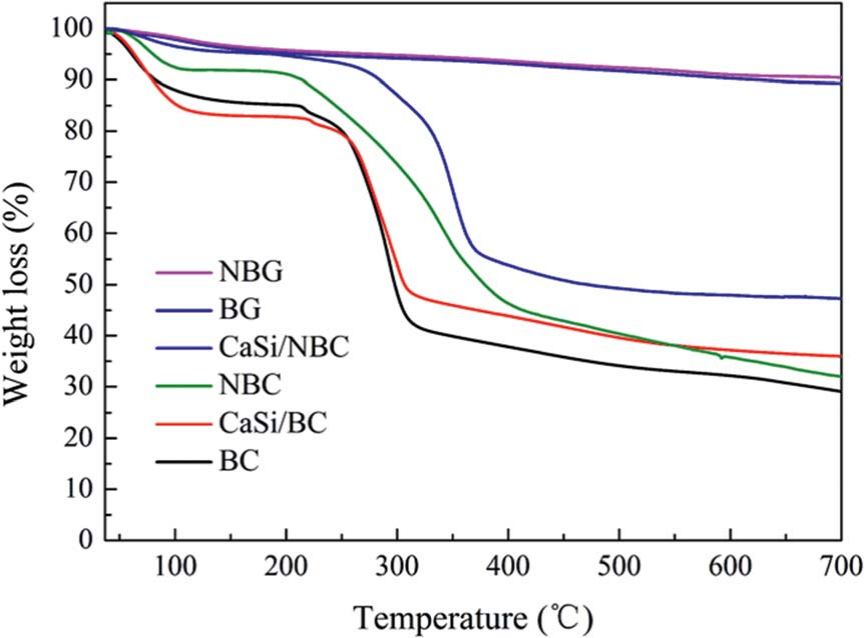
TGA curves of BC, NBC, CaSi/BC, CaSi/NBC, BG and NBG samples.

TGA was used to measure the mass loss of the test samples during heating to 700 °C. From Fig. 7, for the BC, NBC, CaSi/BC, and CaSi/NBC samples, we could observe nearly 4–15% weight loss at around 120 °C, which was due to the loss of adsorbed water, and nearly 5% weight loss at around 250 °C was observed due to the loss of residual crystalline water. A sharp weight loss also occurred at 250–350 °C for these BC-containing samples, which was associated with the splitting of the BC structure and formation of large amounts of volatiles from the decomposed BC units. It is worth noting that BC displayed a greater weight loss of about 58% up to 350 °C when compared with NBC (45%), CaSi/BC (52%), and CaSi/NBC (40%). Impressively, the weight losses of NBC and CaSi/NBC happened at a higher decomposition temperature and less loss trend than those of the samples with no amino-modification (BC and CaSi/BC). This phenomenon could be related to the decomposition of the –NH_2_ groups, which can retard and reduce the decomposition of BC and improve the thermal stabilities of the NBC and CaSi/NBC samples.^48–50^ Furthermore, there was another weight loss step in the range of 350–450 °C for the NBC and CaSi/NBC samples, which was caused by the volatilization of the –NH_2_ groups. These results confirmed again that the –NH_2_ groups had been successfully grafted onto the surface of the BC fibers, and these results were in accordance with the results of FTIR analyses. Furthermore, it could be seen that there was nearly no weight loss after 500 °C for the CaSi/BC and CaSi/NBC samples, indicating that most of the organic components were decomposed. While for the BC and NBC samples, the weight loss was nearly invariable up until 700 °C, and the corresponding residual weights were about 29% and 32% of their original masses observed as char products at 700 °C. The use of a low heating rate and inert nitrogen atmosphere in the TGA testing explained the incomplete volatilization of the BC and NBC samples.^8^ From the TGA curves of CaSi/BC and CaSi/NBC, the residual weights were about 36% and 48%, respectively, which indicated that the amount of BC decreased with an increase in the amount of the CaO/SiO_2_ precursors. This trend was expected since the ceramic can maintain its mass, and all the mass loss was attributed to BC. Furthermore, it is worth noting that the residual weight of CaSi/NBC was 10%, which was more than that of the CaSi/BC scaffold, suggesting that the grafting of –NH_2_ groups onto the BC network can enhance the load capacity of the CaO/SiO_2_ precursors on the BC fibers.

### 3.5 *In vitro* bioactivity study

**Fig. 8:**
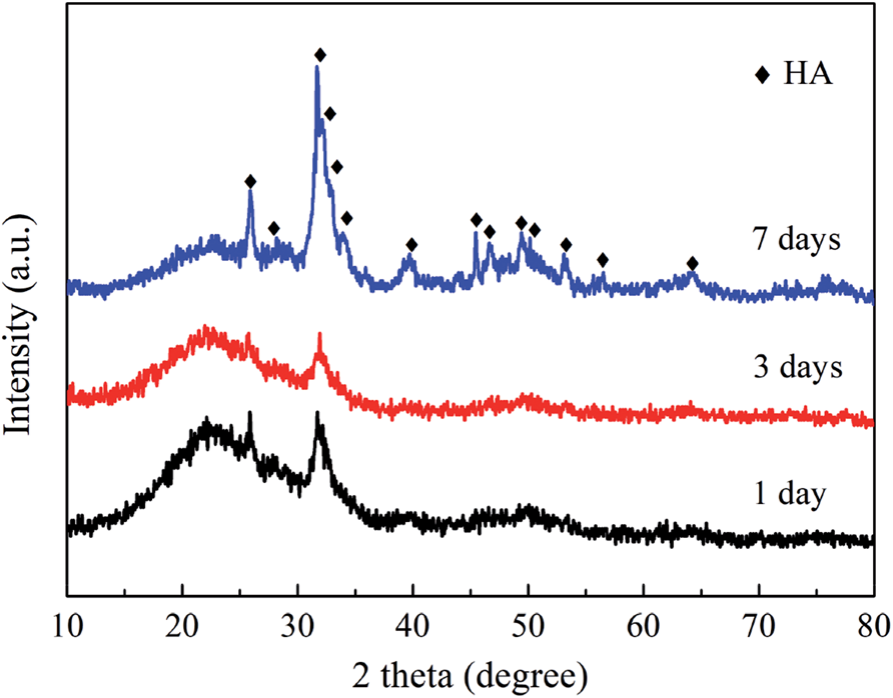
XRD patterns of NBG scaffolds after immersion in SBF for 1, 3 and 7 days.

When immersed in a body fluid, BG can effectively adsorb amorphous calcium phosphate or hydroxyapatite (HA) deposited, which is responsible for its strong bonding with the surrounding tissues.^2,51^Fig. 8 shows the XRD results of the NBG scaffold after being immersed in SBF for 1, 3 and 7 days. As shown in Fig. 8, the characteristic diffraction peaks assigned to HA (PDF#84-1998) are observed in the XRD analyses of all the samples, which can confirm the presence of HA and its successfully deposition on the surface of the BG scaffold. In addition, more characteristic peaks have been clearly observed, and some main peaks become sharp and clear for the sample after 7 days of immersion. For example, the characteristic peaks at 25.9°, 31.8°, 39.8°, 49.5°, and 64.0°, are assigned to the (0 0 2), (1 2 1), (3 1 0), (1 2 3), and (3 0 4) crystal planes of HA, respectively, indicating that the HA becomes more crystalline upon increasing the immersion time.

**Fig. 9:**
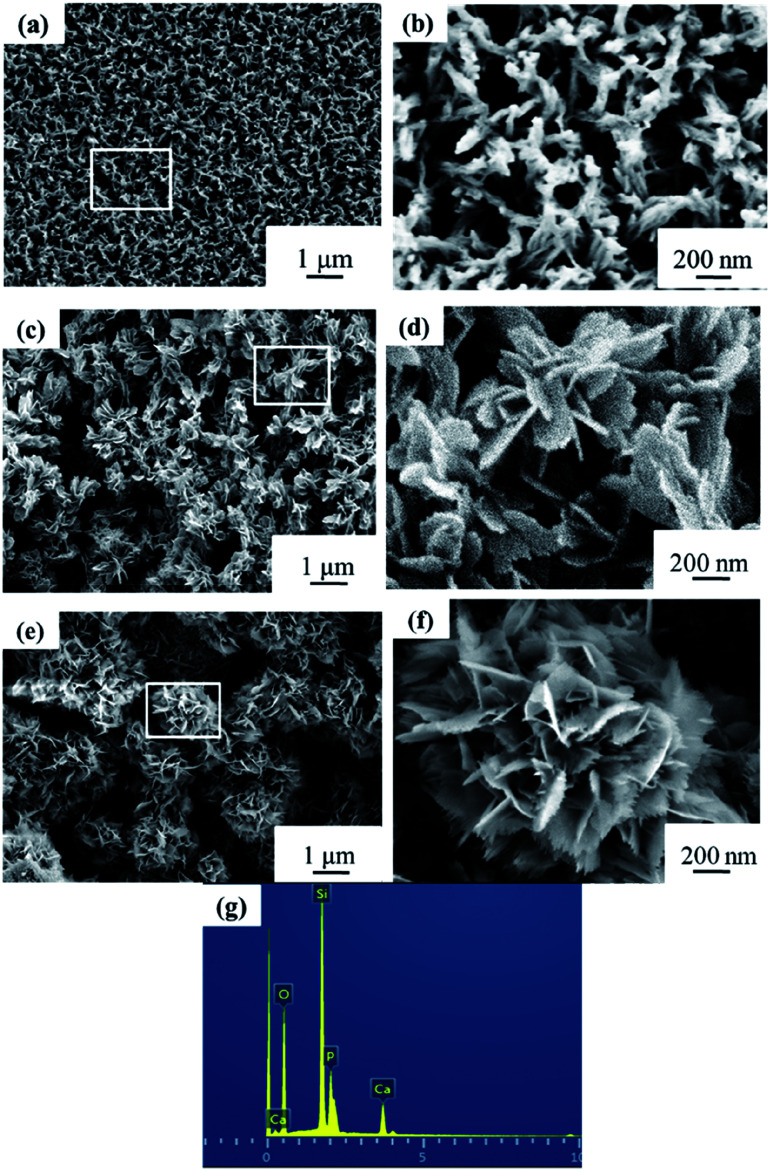
SEM images and the corresponding high magnification images of NBG scaffolds after immersion in SBF for (a and b) 1, (c and d) 3, and (e and f) 7 days (insets show local enlarged areas) and (g) EDS profile of NBG scaffold for 7 days as shown in figure (f).

Fig. 9a–f show the SEM images and corresponding high magnification images of the NBG scaffold after being immersed in SBF for 1, 3 and 7 days. As shown in Fig. 9a and b, the 3D nanofibrous NBG scaffold is covered with a layer of needle-like HA after immersion for only 1 day, indicating that the deposition of the minerals is quite fast. After being immersed in SBF for 3 days, a large amount of HA is observed on the surface of the NBG scaffold, and the morphology changes to an irregular small flower-like structure, which can be related with the growth of HA crystallites upon increasing the immersion time. It is worth noting that almost all the HA crystallites have grown and show a blooming flower-like morphology with many prickly flaky structures after being immersed in SBF for up to 7 days. The EDS profile of the NBG scaffold after 7 days of immersion is also shown in Fig. 9g, which confirms the distribution of the Ca, O, P and Si elements within the HA structure.

It is believed that the speed of mineral deposition is closely related to the bioactivity of a biomaterial.^30,52^ In combination with the SEM and XRD analyses, a fast deposition can be observed on the as-obtained NBG samples in this study, which suggests that the samples have great bioactivity. The results of EDS analyses of the NBG scaffolds after being immersed in SBF for 1, 3, and 7 days are listed in Table 2. Table 2 shows that all the NBG scaffolds consist of Ca, P, Si and O elements after being immersed in SBF for 1, 3 and 7 days, and the contents of Ca and P elements increase upon increasing the immersion time. The atomic ratio of Ca/P increases from 0.91 and 1.18 up to 1.70 after immersion in SBF for 1, 3 and 7 days, which suggests that the samples can quickly absorb Ca and P from the SBF solution and quickly induce the formation of HA thus showing good bioactivity. It is worth noting that the atomic ratio of Ca/P reaches 1.70 after 7 days of immersion, which is close to the theoretical value of HA (Ca_5_(PO_4_)_3_(OH), 1.67), indicating the HA is very well crystallized.

**Table 2:** EDS analyses of NBG scaffold after immersion in SBF for 1, 3, and 7 days

Elements (at%)	Ca	P	Si	O	Ca/P
NBG for 1 day	5.53	6.05	33.79	54.63	0.91
NBG for 3 days	10.39	8.78	25.67	55.16	1.18
NBG for 7 days	35.89	21.11	1.55	41.45	1.70

Based on the current research, the surface chemistry and porous structure of the bioactive material and the released Ca^2+^ from the bioactive materials can control the deposition of HA.^53,54^ In addition, the development of HA crystals on the NBG nanofibers involves two sequential steps, *i.e.*, nucleation and growth.^55^ For the NBG nanofibers and HA nucleation, the smaller the contact angle between them, the more favorable the HA nucleation.^56^ The interconnected porous structure with high specific surface area of the NBG scaffold can greatly reduce the contact angle between HA and the substrate, which makes an important contribution to the rapid deposition of HA in SBF.^57^ In addition, the NBG nanofibers have certain solubilities in the SBF solution. In the process of dissolution, a large amount of Ca^2+^ ions is released during the initial stage of immersion and the relative saturation of HA increases, which can effectively promote the rapid crystallization of HA. Therefore, it is inferred in this study that the NBG scaffolds consist of nano-scaled BG nanofibers and interconnected porous structures and have a very high and stable bioactivity and thus, they show great promise in bone tissue repair engineering.

## 4. Conclusions

In this study, we prepared a 3D nanofibrous BG scaffold using amino-modified BC as the template *via* a facile modified sol–gel technique under ultrasonic treatment. The amino-modified BC scaffold could absorb more CaO and SiO_2_ precursors deposited on its surface than the untreated BC scaffold, which promoted the successful formation of the nanofibrous BG scaffold after being calcined at 700 °C for 3 h. The immersion test in SBF showed that a large amount of HA had been successfully deposited on the surface of the NBG scaffold, and the morphology of HA changed from a needle-like structure to a blooming flower-like structure after being immersed in SBF from 1 to 7 days; this indicated a fast nucleation and growth of the HA crystallites with an increase in the immersed time. These results suggested that the NBG scaffolds obtained using this facile method have a very high bioactivity. This information should be useful for further research and practical applications of this new nanostructured NBG scaffold in biomedical fields, especially for the field of bone repair and regeneration.

## Conflicts of interest

There are no conflicts to declare.

## Supplementary Material

## References

[cit1] Dinarvand P., Seyedjafari E., Shafiee A., Jandaghi A. B., Doostmohammadi A., Fathi M. H., Farhadian S., Soleimani M. (2011). ACS Appl. Mater. Interfaces.

[cit2] Rahaman M. N., Day D. E., Bal B. S., Fu Q., Jung S. B., Bonewald L. F., Tomsia A. P. (2011). Acta Biomater..

[cit3] Wu S., Liu X., Yeung K. W. K., Liu C., Yang X. (2014). Mater. Sci. Eng., R.

[cit4] Wang X. Q., Zhang L., Ke X. R., Wang J. C., Yang G. J., Yang X. Y., He D. S., Shao H. F., He Y., Fu J. Z., Xu S. Z., Gou Z. R. (2015). RSC Adv..

[cit5] Bretcanu O., Misra S. K., Yunos D. M., Boccaccini A. R., Roy I., Kowalczyk T., Blonski S., Kowalewski T. A. (2009). Mater. Chem. Phys..

[cit6] Govindan R., Kumar G. S., Girija E. K. (2015). RSC Adv..

[cit7] Li X., Wang X., Chen H., Jiang P., Xiaoping Dong A., Shi J. (2007). Chem. Mater..

[cit8] Busuioc C., Stroescu M., Stoica-Guzun A., Voicu G., Jinga S.-I. (2016). Ceram. Int..

[cit9] Gao C., Gao Q., Bao X., Li Y., Teramoto A., Abe K. (2011). J. Am. Ceram. Soc..

[cit10] Wan Y. Z., Huang Y., Yuan C. D., Raman S., Zhu Y., Jiang H. J., He F., Gao C. (2007). Mater. Sci. Eng., Proc. Conf..

[cit11] Baino F., Fiorilli S., Vitale-Brovarone C. (2016). Acta Biomater..

[cit12] Ondarcuhu T., Joachim C. (1998). Europhys. Lett..

[cit13] Feng L., Li S. H., Li H. J., Zhai J., Song Y. L., Jiang L., Zhu D. B. (2002). Angew. Chem., Int. Ed..

[cit14] Ma P. X., Zhang R. Y. (1999). J. Biomed. Mater. Res..

[cit15] Hong Y., Chen X., Jing X., Fan H., Gu Z., Zhang X. (2010). Adv. Funct. Mater..

[cit16] Kim H. W., Lee H. H., Knowles J. C. (2006). J. Biomed. Mater. Res., Part A.

[cit17] Wang J., Wan Y. Z., Luo H. L., Gao C., Huang Y. (2012). Mater. Sci. Eng., C.

[cit18] Dou Y., Wu C., Chang J. (2012). Acta Biomater..

[cit19] Deliormanlı A. M. (2015). Ceram. Int..

[cit20] Kim M., Kim G. (2015). J. Colloid Interface Sci..

[cit21] Liverani L., Roether J. A., Nooeaid P., Trombetta M., Schubert D. W., Boccaccini A. R. (2012). Mater. Sci. Eng., A.

[cit22] Echezarreta-López M. M., de Miguel T., Quintero F., Pou J., Landín M. (2017). Biomater. Appl..

[cit23] Li W., Garmendia N., Pérez de Larraya U., Ding Y., Detsch R., Grünewald A., Roether J. A., Schubert D. W., Boccaccini A. R. (2014). RSC Adv..

[cit24] Grande C. J., Torres F. G., Gomez C. M., Bano M. C. (2009). Acta Biomater..

[cit25] Hong L., Wang Y. L., Jia S. R., Huang Y., Gao C., Wan Y. Z. (2006). Mater. Lett..

[cit26] Saboori A., Rabiee M., Moztarzadeh F., Sheikhi M., Tahriri M., Karimi M. (2009). Mater. Sci. Eng., Proc. Conf..

[cit27] Wu M., Wang Q., Liu X., Liu H. (2013). Carbon.

[cit28] Klemm D., Schumann D., Udhardt U., Marsch S. (2001). Prog. Polym. Sci..

[cit29] Svensson A., Nicklasson E., Harrah T., Panilaitis B., Kaplan D. L., Brittberg M., Gatenholm P. (2005). Biomaterials.

[cit30] Luo H., Ji D., Li W., Xiao J., Li C., Xiong G., Zhu Y., Wan Y. (2016). Mater. Chem. Phys..

[cit31] Huang X., Zhan X., Wen C., Xu F., Luo L. (2017). J. Mater. Sci. Technol..

[cit32] Stenstad P., Andresen M., Tanem B. S., Stenius P. (2007). Cellulose.

[cit33] Ma Z., Guan Y., Liu H. (2005). J. Polym. Sci., Part A: Polym. Chem..

[cit34] Wen C., Zhan X., Huang X., Xu F., Luo L., Xia C. (2017). Surf. Coat. Technol..

[cit35] Jansen K. A., Atherton P., Ballestrem C. (2018). Semin. Cell Dev. Biol..

[cit36] Wang W., Bai Q., Liang T., Bai H., Liu X. (2017). Int. J. Biol. Macromol..

[cit37] Lai C., Zhang S. J., Wang L. Q., Sheng L. Y., Zhou Q. Z., Xi T. F. (2015). J. Mater. Chem. B.

[cit38] Hammonds R. L., Harrison M. S., Cravanas T. C., Gazzola W. H., Stephens C. P., Benson R. S. (2012). Cellulose.

[cit39] Zhong Z., Qin J., Ma J. (2015). Mater. Sci. Eng., C.

[cit40] Ramani D., Sastry T. P. (2014). Cellulose.

[cit41] Shi S., Chen S., Zhang X., Shen W., Li X., Hu W., Wang H. (2009). J. Chem. Technol. Biotechnol..

[cit42] Ghorbani M., Nowee S. M., Ramezanian N., Raji F. (2016). Hydrometallurgy.

[cit43] Wen C., Guan S., Peng L., Ren C., Wang X., Hu Z. (2009). Appl. Surf. Sci..

[cit44] Luo H., Xiong G., Zhang C., Li D., Zhu Y., Guo R., Wan Y. (2015). Mater. Sci. Eng., C.

[cit45] Sun X., Yang L., Li Q., Zhao J., Li X., Wang X., Liu H. (2014). Chem. Eng. J..

[cit46] Shankhwar N., Kumar M., Mandal B. B., Srinivasan A. (2016). Mater. Sci. Eng., C.

[cit47] Shankhwar N., Kothiyal G. P., Srinivasan A. (2015). RSC Adv..

[cit48] Hernández-Morales V., Nava R., Acosta-Silva Y. J., Macías-Sánchez S. A., Pérez-Bueno J. J., Pawelec B. (2012). Microporous Mesoporous Mater..

[cit49] Luo S., Xu X., Zhou G., Liu C., Tang Y., Liu Y. (2014). J. Hazard. Mater..

[cit50] Shen J., Huang W., Wu L., Hu Y., Ye M. (2007). Composites, Part A.

[cit51] Fu Q., Rahaman M. N., Fu H., Liu X. (2010). J. Biomed. Mater. Res., Part A.

[cit52] Maheswaran A., Hirankumar G., Karthickprabhu S., Bella R. S. D. (2012). J. Alloys Compd..

[cit53] Takadama H., Kim H.-M., Kokubo T., Nakamura T. (2001). Sci. Technol. Adv. Mater..

[cit54] Ohtsuki C., Kokubo T., Yamamuro T. (1992). J. Non-Cryst. Solids.

[cit55] Juhasz J. A., Best S. M., Auffret A. D., Bonfield W. (2008). J. Mater. Sci.: Mater. Med..

[cit56] Cerruti M. G., Greenspan D., Powers K. (2005). Biomaterials.

[cit57] Cerruti M., Greenspan D., Powers K. (2005). Biomaterials.

